# Trends in global dependency on the Indonesian palm oil and resultant environmental impacts

**DOI:** 10.1038/s41598-020-77458-4

**Published:** 2020-11-26

**Authors:** Yosuke Shigetomi, Yuichi Ishimura, Yuki Yamamoto

**Affiliations:** 1 Faculty of Environmental Science, Nagasaki University, 1-14 Bunkyo-machi, Nagasaki, 852-8521 Japan; 2 Faculty of Economics, Kindai University, 3-4-1 Kowakae, Higashi Osaka, Osaka 577-8502 Japan

**Keywords:** Environmental impact, Sustainability

## Abstract

Rapid growth in the international demand for palm oil has triggered considerable global concern because oil palm plantations deteriorate the environment where they are developed, resulting in complex environmental impacts in the producer nations. Here, we illustrate the historical trends in the structure of Indonesian palm oil supply chains and how these have been affected by the final demand of other nations since 2000 by using the most recent dataset of global material flows of palm oil and a global input–output database. In addition, the combination of spatial land-use change with palm oil consumption along the supply chains illustrates the linkages between ultimate consumption and land-use changes due to the palm oil plantations. As a result, the major contributors to palm oil production in Indonesia were mostly stable, being India, China, Western Europe, the United States, and Japan. However, the contribution of Indonesia declined by 6% during 2000–2013, illustrating a possible shift towards palm oil being used for non-food demands, such as apparel and medicines. Building on consumption-based accounting schemes as demonstrated by this study are considered necessary to protect local ecosystems and society.

## Introduction

Palm oil is an indispensable resource for daily commodities, and is widely used in margarine, shortening, chocolates and snacks, and packaged and fast food. The main advantage of palm oil as an edible oil is its low price and high utility compared with the other vegetable oils^1,2^. It is also utilized to produce commodities that are not related to food, such as cosmetic products, soaps and detergents, grease, and printer ink. In addition, palm oil is useful for biodiesel production, which has become increasingly important given the role of fossil fuels in greenhouse gas (GHG) emissions. The demand for palm oil is therefore expected to increase markedly in the future, with this increase underpinned by the general versatility of the oil for producing daily goods and its potential role in climate change mitigation^3–5^.

Although the provenance of oil palm production used to be Africa, the production of oil palm, as a primary crop has shifted to South East Asia, particularly Indonesia and Malaysia. Surpassing Malaysia in 2008, Indonesia has experienced a rapid expansion of oil palm plantations in recent decades, and the country was responsible for almost 50% of global palm oil production in 2017; Indonesia is now the world’s largest producer^6^. Consequently, there is an emerging consensus that the palm oil industry has benefited the rural Indonesian economy considerably in terms of improving household welfare and local infrastructure^7^. This consensus has arisen because, compared to conventional crops such as rubber and rice, oil palm cultivation has higher returns in terms of land and labor requirements. This higher crop productivity appears to be associated with increases in farmers’ income^8^. However, the demand for palm oil has been a driving force behind massive environmental and social concerns related to the land-use change (LUC) associated with the expansion of oil palm plantations in the producer countries. This LUC has been shown to increase deforestation^9^, biodiversity loss^10^, forest fires and air pollution^11^, carbon emissions^12^, water abstraction^13^, and land conflicts^14^. Further, fires in oil palm areas are common in areas managed by large plantations or smallholders^15^, and occur even in the concession areas^11^. The forest fires and deforestation due to the expansion of plantations cause considerable GHG emissions^16,17^, and the atmospheric pollution attributed to these fires affects the health of humans in the surrounding areas and even neighboring states^11^. However, most palm oil consumption takes place outside Indonesia; for example, 85% of the total palm oil produced in 2018 was exported to other nations^6^. Consequently, supply chain management and a shift towards sustainable palm oil production has gained increasing attention through initiatives such as the Roundtable on Sustainable Palm Oil (RSPO)^18–20^. However, the quantities exported, as mentioned above, do not fully account for the palm oil that is utilized to produce the intermediates of commodities that are finally consumed via international supply chains; e.g., production of palm oil in Indonesia to be utilized for washing textiles in China, which are then exported as clothing to Japan. Given the high versatility of palm oil, it is necessary to quantitatively trace this upstream utilization of palm oil which is consumed by nations other the producer nation in order to steer supply chain management towards sustainable production and consumption as set out in the UN Sustainable Development Goals (SDGs)^21^.

The aim of this study was to estimate how much nations depend ultimately on the palm oil in the international supply chains, and their dependencies drive environmental impacts in the producer nation. To address these issues, we employed an input–output analysis using a global multi-regional input–output model (MRIO) in line with consumption-based accounting^22,23^. This approach has been a powerful tool for estimating direct and indirect goods and services requirements, and for examining the socio-environmental pressures associated with international trade and meeting final demands^24,25^. Such direct and indirect environmental pressures are often referred to as ‘footprints’, and numerous footprint studies have been conducted on GHGs^26,27^, water use^28,29^, material use^30,31^, air pollutants^32^, and other indicators. Further, extensive analyses have examined consumption-based environmental impacts in the producer nation(s) of global resource utilization through the global supply chains. For example, Moran et al.^33^ investigated how final consumption by nations drives biodiversity loss in the producing nations; for example, nickel mining in New Caledonia, coltan in the Democratic Republic Congo, cut flowers in Kenya, and forestry in Papua New Guinea. Their approach is based on Lenzen et al.^34^, which is considered to be the pioneering study for estimating the number of species threatened (i.e. biodiversity loss) by international trade. In addition, they highlighted the linkage between consumer and producer countries via global supply chains and the biodiversity footprint. Nakajima et al.^35^ estimated the areas of LUC at nickel mining sites around the world and how these changes were induced by global final consumption in the impacted nations. Verones et al.^36^ examined the biodiversity footprints associated with global water use, LUC, and GHG emissions and how these were affected by final consumption. In that study, they used a combination of the MRIO with life cycle impact assessment (LCIA) and considered the consequences of resource consumption on ecosystems. Többen et al.^37^ also analyzed the impact of biodiversity on global consumption of the four major oil crops, i.e., soybean, rapeseed, sunflower, and oil palm. For the biodiversity footprint associated with palm oil consumption, they found that Western Europe and Australia were hotspots given their high per-capita footprints, compared to the other economically developed nations, such as the United States and Japan.

Against this backdrop, this study has a similar focus as that of Nakajima et al.^35^ and Többen et al.^37^, in that it attempts to clarify the structure of consumption-based palm oils produced in Indonesia, and assesses the associated LUC for the producer nation. We aim to highlight these historical trends since 2000 rather than providing a snapshot for a single year by using the most recent public dataset and spatial data currently available for oil palm plantations^38^. In addition, we focus on the contributions of household consumption on each of the nations in order to understand the linkage between daily lifestyle and Indonesian palm oil production. Finally, we discuss the policy implications associated with sustainable Indonesian palm oil consumption and production, as well as future research.

## Results

### Structure of the Indonesian palm oil footprint by final demand

**Figure 1:**
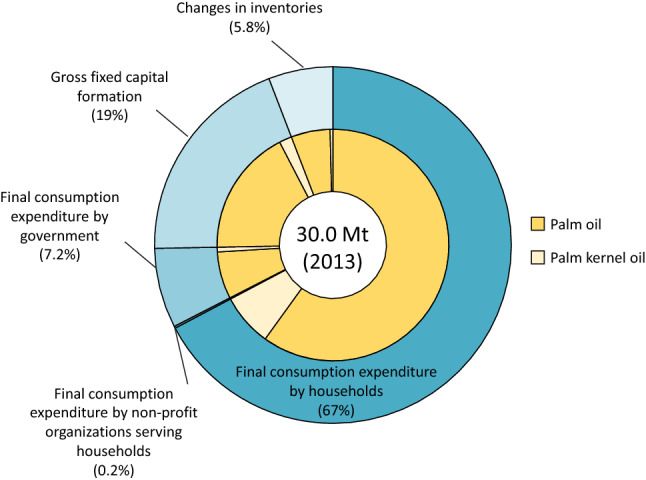
Composition of the global Indonesian palm oil footprint in 2013.

Material flow analysis of both palm oil (PO) and palm kernel oil (PKO) (hereafter, these two oils are collectively referred to as palm oil) produced in Indonesia revealed that the domestic production accelerated from 13.3 to 19.0 million tons per year (i.e., Mt/yr) between 2005 and 2006, and that it kept increasing until 2013. In that period, the export of palm oil from Indonesia was boosted from 14.6 to 24.8 Mt/yr, which accounted for 77% and 83% of the total global palm oil production in 2006 and 2013, respectively. In response to an increase in Indonesian palm oil production, the global Indonesian palm oil footprint (PF) also increased from 7.7 to 30.0 Mt/yr. Figure 1 illustrates the composition of the global Indonesian PF in 2013 for each oil type and final demand.

The outer grey-green layer of the figure shows that household consumption was the largest contributor among the global final demands, and that it accounted for 67% of the total Indonesian PF in 2013. The second largest contributor was gross fixed capital formation, which was responsible for 22% of the total Indonesian PF. Compared to 2000, which is the initial year of analysis, the contribution of gross fixed capital formation increased by 7.7% compared to household consumption, which decreased by 8.6%, implying that utilization of palm oil is gradually becoming more important for infrastructure. The third largest contributor was government (7.2%), followed by changes in inventories and valuables (5.8%) and non-profit organizations serving households (0.2%). Figure 1 also shows the relative compositions of PO and PKO compared to the total PF as shown by the inner orange layer of the figure. For the final demands of all oil types, palm oil was the main source of the PF, accounting for approximately 90% of the total PF during the period, while the contributions of PKO varied between 2 and 9% during 2000–2013. This implies that the global demand of non-food commodities that utilize PKO have gradually increased as consumption patterns have changed.

By detailing the sectoral contributions (hereafter, the sector name is written in *italic*), *manufacture of food products* and *beverages and tobacco products* were dominant in the Indonesian PF, accounting for 2.3 Mt/yr in 2000, which is equivalent to 30% of the total Indonesian PF. Other sectors included *manufacture of chemical and chemical products* (1.1 Mt/yr), *construction* (0.56 Mt/yr), and *manufacture of textiles,* and *wearing apparel and leather products* (0.46 Mt/yr). Despite decreasing after 2005 and 2009, the *manufacture of food products, beverages and tobacco products* sector was consistently the largest driver of the Indonesian PF, accounting for approximately 8.7 Mt/yr (29%) in 2013. The PF for *construction* was estimated to be 3.6 Mt/yr, increasing from 7.1% to 12%. In addition, *human health and social work activities* (1.2 Mt/yr) and *manufacture of coke and refined petroleum products* (0.87 Mt/yr) also induced a considerable portion of the Indonesian PF, accounting for the fifth and eighth largest contributions to the PF in 2013. In contrast, *manufacturing of chemicals and chemical products* and *manufacture of textiles, wearing apparel and leather products*, which are mentioned above, contributed less to the total PF in 2013 than they did in 2000, although both of their PF increased (3.1 Mt/yr and 1.2 Mt/yr, respectively).

### Trends in the Indonesian palm oil imports and footprints per nation

The 10 largest importer nations of Indonesian palm oil during 2000–2013, along with the rest of the world (ROW), are listed in Table 1 (a). The import shares of the major nations dominate for total Indonesian palm oil imports, accounting for 81% in 2000 and 63% in 2013. This implies that most nations depend indirectly on Indonesian palm oil. India has been the largest palm oil importer since 2000, with the most recent imports amounting to 6.9 Mt/yr in 2013. China was the second largest importer in 2013, boosting their palm oil imports almost sixfold, from 0.54 Mt/yr to 3.1 Mt/yr during the analyzed period. The Netherlands used to be the second largest importer in 2000 and they remained the largest importer in the EU region until 2013. Also, compared to other nations in the early 2000s, German imports increased markedly before dropping towards 2013, while Italian and Spanish imports increased. More recently, importing nations have consisted primarily of the United States, Russia, Turkey, and Brazil. The rest of the 193 nations and regions (i.e., 236 nations and regions listed the trade data – 43 nations included in the MRIO employed in this study); i.e., ROW, showed the highest growth in 2013 compared to the 2000 level, implying that the Indonesian palm oil trade has become more multilateral.

**Table 1:** Total amount of palm oil imports and palm oil footprints from Indonesia, and the 10 nations with the largest palm oil imports (a) and footprints (b) in each year of 2000–2013. Unit: 10^3^ ton (kt).

(a)														
Rank	2000		2001		2002		2003		2004		2005		2006	
1	2077	India	1929	India	2298	ROW	2670	India	3312	ROW	4406	ROW	5626	ROW
2	1008	Netherlands	1412	ROW	2139	India	2033	ROW	3281	India	3037	India	3044	India
3	875	ROW	1124	Netherlands	1571	Netherlands	1000	China	1376	China	1737	China	2292	China
4	543	China	509	China	608	China	958	Netherlands	1318	Netherlands	1625	Netherlands	1833	Netherlands
5	186	Germany	268	Turkey	255	Germany	218	Germany	301	Germany	406	Germany	451	Germany
6	167	Spain	265	Germany	243	Spain	204	Turkey	210	Spain	283	Turkey	387	Turkey
7	113	Turkey	231	Spain	216	Turkey	174	Spain	202	Italy	184	Italy	201	Italy
8	76	USA	100	Mexico	54	Italy	79	Italy	96	USA	165	Spain	194	Spain
9	50	Mexico	87	Italy	40	Mexico	56	Russia	69	Turkey	89	Russia	164	Russia
10	50	Italy	50	Russia	35	France	19	Greece	46	Russia	69	UK	155	Brazil
	5289	World total	6128	World total	7643	World total	7514	World total	10,401	World total	12,270	World total	14,673	World total

Rank	2007		2008		2009		2010		2011		2012		2013	
1	5435	ROW	5707	India	6333	India	6172	India	7376	ROW	7962	ROW	8427	ROW
2	1772	India	5280	ROW	5541	ROW	5983	ROW	6017	India	6469	India	6942	India
3	1386	Netherlands	2290	China	3311	China	2727	China	2622	China	3765	China	3104	China
4	834	China	1926	Netherlands	1860	Netherlands	1640	Netherlands	1372	Netherlands	1876	Netherlands	1909	Netherlands
5	661	Germany	566	Italy	846	Italy	792	Italy	681	Italy	804	Italy	1222	Italy
6	388	Turkey	482	Germany	537	Germany	446	Germany	429	Spain	416	Russia	744	Spain
7	285	Italy	271	Spain	419	Spain	409	Spain	379	Russia	342	Spain	534	USA
8	207	Russia	188	Brazil	191	Brazil	304	Brazil	315	Germany	333	Brazil	506	Russia
9	156	Spain	130	USA	164	USA	280	Russia	297	Brazil	311	Turkey	390	Turkey
10	125	USA	107	Turkey	91	Turkey	93	Turkey	96	Turkey	270	Germany	380	Brazil
	11,614	World total	17,194	World total	19,645	World total	19,094	World total	19,885	World total	22,867	World total	24,846	World total

(b)														
Rank	2000		2001		2002		2003		2004		2005		2006	
1	1694	India	1659	Indonesia	1857	ROW	2511	Indonesia	2858	India	3546	ROW	4300	ROW
2	1314	Indonesia	1618	India	1738	India	2133	India	2682	ROW	2713	India	2592	Indonesia
3	1001	ROW	1300	ROW	1723	Indonesia	1811	ROW	1303	China	1563	China	2353	India
4	615	USA	761	USA	873	USA	1126	China	1245	Indonesia	804	Indonesia	2154	China
5	593	China	663	China	808	China	848	USA	638	USA	610	Germany	1630	USA
6	360	Germany	437	Germany	479	Germany	372	Germany	487	Germany	603	USA	686	Germany
7	295	Netherlands	335	Japan	350	Japan	370	Japan	354	Netherlands	427	Netherlands	626	Japan
8	281	Japan	268	Netherlands	301	UK	233	UK	329	Japan	363	UK	490	UK
9	182	Spain	241	Spain	301	Netherlands	225	Netherlands	321	Italy	336	Italy	441	Turkey
10	179	UK	224	UK	283	France	224	Spain	275	Spain	334	Japan	434	Italy
	7,718	World total	9,179	World total	10,595	World total	11,524	World total	12,112	World total	13,321	World total	19,033	World total

Rank	2007		2008		2009		2010		2011		2012		2013	
1	4557	ROW	4801	India	5615	India	5386	ROW	6043	ROW	6959	ROW	7345	ROW
2	4078	Indonesia	4583	ROW	4937	ROW	4960	India	4833	India	4914	India	5136	India
3	1658	India	2131	China	3108	China	3258	Indonesia	3432	Indonesia	4127	China	3687	China
4	1625	USA	1605	Indonesia	1481	Indonesia	2904	China	3117	China	3803	Indonesia	3202	Indonesia
5	1426	China	1058	USA	867	USA	1421	USA	1539	USA	1757	USA	2050	USA
6	678	Germany	733	Germany	804	Germany	681	Germany	663	Germany	762	Germany	991	Germany
7	639	Japan	629	Italy	798	Germany	668	Italy	649	Italy	723	Japan	857	Italy
8	499	Turkey	399	Japan	475	Spain	551	Japan	640	Japan	624	Italy	691	Japan
9	454	Italy	391	Spain	383	Japan	509	Brazil	550	Brazil	596	Brazil	677	Russia
10	448	UK	381	France	379	UK	433	Spain	504	Russia	591	Russia	649	Brazil
	19,485	World total	19,602	World total	21,607	World total	24,321	World total	25,700	World total	28,935	World total	29,957	World total

The top 10 nations that contribute most to the Indonesian PF are presented in Table 1 (b). These nations did not vary markedly during 2000–2013, and India and China were still two of the largest contributors towards the Indonesian PF. The Indonesian PF induced by India and China were estimated to be from 1.7 to 5.2 Mt/yr and from 0.60 to 3.8 Mt/yr between 2000 and 2013, respectively. Thus, the increase in the rate of China’s contribution to the PF was more than twice that of India’s. In 2000, Indonesia had the second highest PF (1.0 Mt/yr). Due to increased domestic consumption, Indonesia had the third largest PF in 2013. The United States was ranked as the third largest contributor to the Indonesian PF in 2000, and the fourth largest contributor behind Indonesia in 2013. Other nations that contributed to Indonesian PF in 2013 were Germany (0.99 Mt/yr), Italy (0.85 Mt/yr), Japan (0.69 Mt/yr), Russia (0.67 Mt/yr), Brazil (0.65 Mt/yr), and Spain (0.60 Mt/yr). Importantly, most nations’ PF exceeded their palm oil import from Indonesia. For example, Japan’s imports of Indonesian palm oil was 0.13 Mt/yr in 2013, which was less than the 10 largest importer nations (Table 1(a)). However, the PF of Japan was the seventh highest, exceeding their palm oil import and PF by more than fivefold. Indeed, the PF of 38 out of 43 nations and the ROW exceeded their imports, indicating that most nations depend largely on hidden palm oil consumed via the international supply chains. The exceptions are India, the Netherlands, Italy, and Spain along with ROW, which are considered to be hubs for products related to Indonesian palm oil.

### Trends in the palm oil footprints induced by household daily consumption

**Figure 2:**
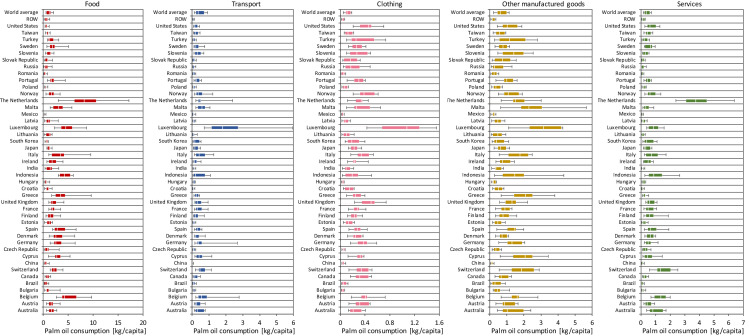
Trends in the five sectoral contributions of the per-capita Indonesian palm oil footprints for household consumption by 43 countries, ROW regions, and world average during 2000–2013. The bars indicate the maximum and minimum per-capita footprints. The boxes represent 25th percentile (left hinge), median and 75th percentile (right hinge).

The per-capita Indonesian PF induced by household consumption across nations is shown in Fig. 2, which illustrates the trends in different sectors and how they affect the total PF. In the figure, the commodities are aggregated into five categories; food, transport, clothing, other manufactured goods (e.g. cosmetics), and services. For the total amounts, the global average per-capita PF for household consumption varied from 1.3 kg to 4.2 kg during 2000–2013. In the latest year, the largest total per-capita PF was the Netherlands (17.9 kg/yr), followed by Luxemburg (16.5 kg/yr), Malta (12.9 kg/yr), and Italy (11.4 kg/yr). Many other European nations had higher per-capita PF than the world average, while most of those in Eastern Europe, Asia, and the Americas had lower per-capita PF, except Indonesia, Australia, and the United States, respectively. Compared to the per-capita PF of Indonesia, the other nations with high national PF, such as India and China, had very small per-capita PF due to their populations being more than five-times that of the Indonesian population. In other words, domestic consumption of palm oil plays an important role in Indonesian households, mainly in terms of food demand, even when the downstream consumption outside of Indonesia is considered.

Unsurprisingly, household food demand was a major driver of the per-capita PF in all of the nations. However, a broad range of per-capita footprints related to food emerged among nations. The per-capita footprints for food in the Netherlands were estimated to be between 3.0 and 17.1 kg, while those in Romania, which had one of the lowest per-capita PF, ranged between 0.09 and 0.81 kg. Thus, among the ROW, the nations of Western Europe had larger food footprints. A high per-capita footprint for transport was observed for Luxemburg, indicating that the people depend highly on palm oil for biodiesel production, which is used to power automobiles and planes. The per-capita PF for clothing was also the highest in Luxemburg. These two trends are underpinned by the very high income per capita in Luxemburg. Both the United Kingdom and the United States had high per-capita PF for clothing. For other manufactured goods, besides Luxemburg, the per-capita PFs for some of the Mediterranean countries such as Malta, Cyprus, and Greece were also high. Finally, the per-capita PF for services, including housing and medicals, were high in Switzerland and the Netherlands. Compared to the other sectors, the per-capita footprints for this sector in Australia were also high relative to other nations.

### Indirect contributions to land-use changes in Indonesia by nation during 2000–2010

**Figure 3:**
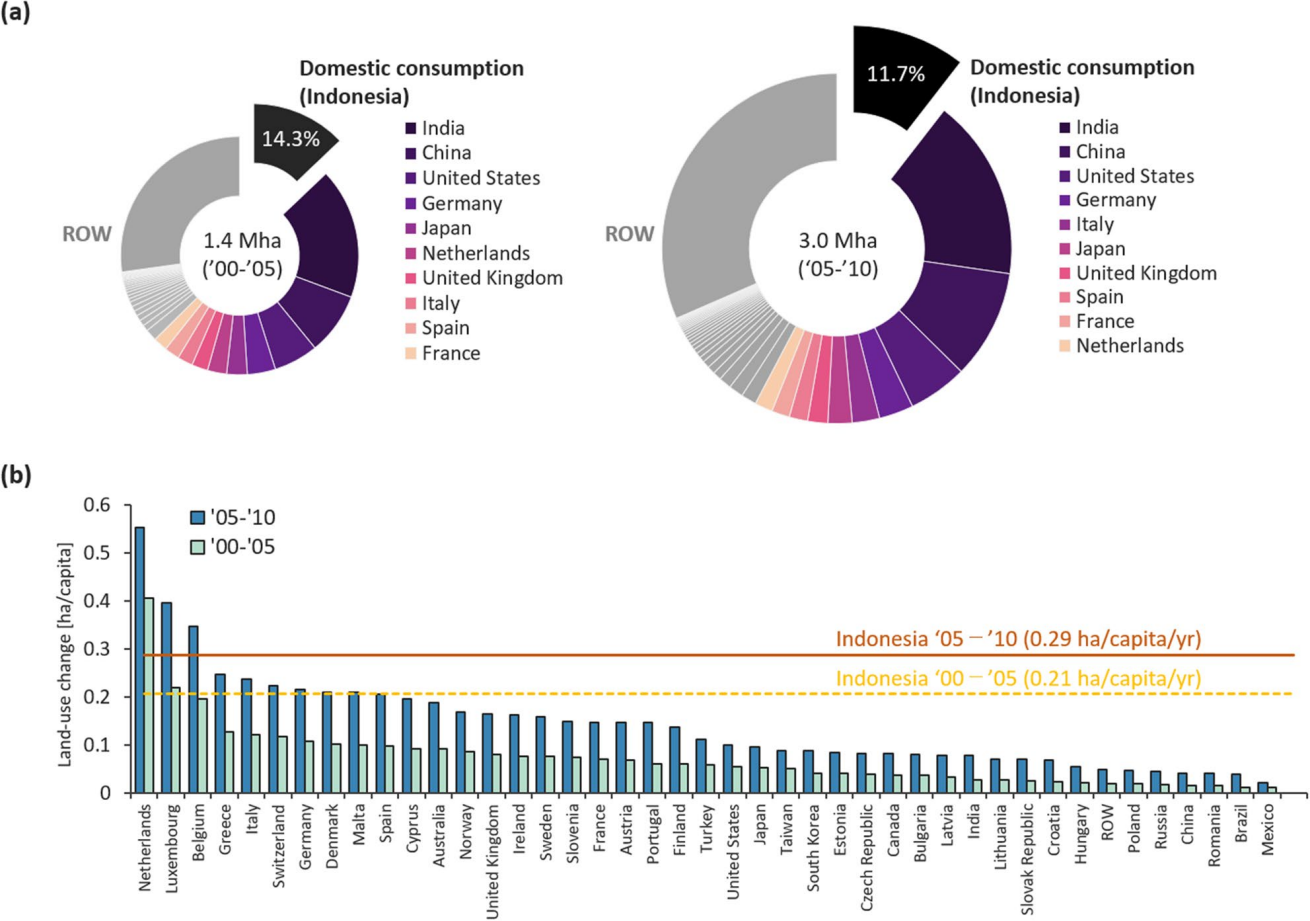
Contributions to the total LUC in Indonesia due to (**a**) total final consumption and (**b**) per-capita contributions to those due to household consumption by 43 nations and the ROW during 2000–2005 and 2005–2010, respectively. The pie chart in figure (**a**) represents the LUC footprints in Indonesia, in descending order, for the 20 largest entire footprints induced by other nations (the 10 largest are colored). The bars in figure (**b**) represent the mean per-capita LUC footprints associated with household consumption in descending order for each nation, except Indonesia. The dashed and solid lines denote the domestic (i.e. Indonesian) contributions during 2000–2005 and 2005–2010, respectively.

The LUC derived from the oil palm plantation in Indonesia was estimated to be 1.4 and 3.0 Mha between 2000–2005 and 2005–2010^38^, respectively. Comparisons of LUC footprints by nation between the two analytical periods are shown in Fig. 3.

In total, almost 86–88% of the LUC footprints were induced by the final consumption outside Indonesia for the decade. The consumption of the top 10 nations, excluding Indonesia and the ROW contributed to approximately half of the total LUC during the two periods (Fig. 3 (a)). On the one hand, between the two periods, India, China, the United States, Germany, and the United Kingdom maintained their rankings as having the first to fourth, and seventh largest LUC footprints. On the other hand, the ranking of Japan and the Netherlands dropped from fifth and sixth to sixth and tenth, respectively. In contrast, the rankings of Italy, Spain, and France increased. The impact of China and Italy on the LUC footprint between the two periods increased by more than three times, which is considered to be high among these top 10 nations. The highest growth rate in the LUC footprint was that of Brazil which increased 3.8-fold.

When focusing on the mean impact of household consumption on the LUC footprint, as shown in Fig. 3 (b), the Netherlands was the largest contributor during both periods, representing 0.41 and 0.55 ha, respectively. At 0.04 and 0.07 ha, these per-capita footprints were approximately tenfold and eightfold larger than the global average, respectively. Besides the Netherlands, compared to affluent nations such as the United States, Japan, and Canada, most western European nations had high per-capita LUC footprints. In particular, the LUC footprints of Greece, Italy, and Norway increased markedly among these nations between the two periods.

Finally, the areas affected by forest fires that were induced by the consumption of the analyzed nations were estimated by combining the LUC results above and satellite data of Indonesian fires^39^. Assuming that fires observed in areas where the oil palm plantations were developed after 2005 are considered, in this study, to be plantations associated with LUC, then we estimate that 0.61 Mha of the areas were destroyed by fire during 2005–2010, i.e., the period over which this study was conducted and that was covered by the fire database. Then, the contribution of final consumption to the fires could be allocated with respect to the PF for each nation (e.g., 19% of the fires were induced by the EU, 17% by India, and 5% by the United States). This environmental information may provide nations with a more comprehensive understanding of their overall consumption, compared to just the consumption of palm oil.

## Discussion

This study quantified historical trends in upstream Indonesian palm oil consumption via the international supply chain; i.e., Indonesian PF and the structural changes by nation. The Indonesian PFs for most of the nations outside Indonesia exceeded the imports of the palm oil, indicating that the amount of direct imports is insufficient for capturing both the nations’ dependence on, and consumer responsibilities on, the consequential environmental impacts as shown in Fig. 3. The findings showed that consumption in India, China, Western Europe, the United States, and Japan have been most responsible for the Indonesian PF since 2000, which is generally consistent with the results of studies on biodiversity loss which have occurred in response to international consumption of food and palm oil for the single analyzed year^37,40^. In addition, the contribution of the ROW, which includes most developing nations (i.e., the 43 analyzed nations generated more than 85% of global GDP in 2008^41^), has been increasing markedly over time. However, the per-capita PF associated with households did not increase significantly, implying that the population growth in the developing nations will be more crucial than lifestyle changes in nations underlying the recent economic situation.

The global Indonesian PF related to food commodities (including accommodation and food service activities) accounted for 34% of the total PF in 2000, implying that non-food consumption of the nations was dominated by the PF. After 2000, the proportion of the PF for non-food consumption to the total fluctuated from 66%, dropping sharply during 2004–2005 and in 2009 by 38%, 31% and 39%, respectively. Since 2009 it increased again and finally reached 67% in 2013, which is a slight increase of 0.8% compared to 2000. Thus, no clear trend in the PF for non-food consumption was observed. However, non-food consumption through existing supply chains is not negligible in so far as reducing the Indonesian PF is concerned. In addition to chemical products, such as soaps and cosmetics, consumption by medical services and apparel are considered to be among the essential contributors to the non-food PF in this study. These demands are expected to increase in a society with an aging population and fast fashion. Changing fast fashion business practices and the consumption behavior of apparel consumers is therefore necessary in order to reduce the environmental impacts associated with the Indonesian palm oil production in the fashion industry^42^. In addition, although it is more difficult to address medical demands compared to apparel demands, encouraging healthier living is expected to play an important role in the indirect reduction of the PF. For example, not only restricting excessive calorie intake, but also promoting walking or cycling can improve people’s health, affecting medical demands indirectly^43^. These changes in habits can also produce the synergetic effects of both improving health and reducing the PF through minimizing transportation. It is also better for both health risks (e.g. cardiovascular disease) and for decreasing the PF to avoid eating processed foods and confectionaries made of palm oil as it is a highly saturated fat^44^.

At a per-capita level, household consumption of palm oil by the BRIC nations, except India, increased rapidly; their PF increased by more than sixfold from 2000 to 2013, while the Indonesian domestic PF only increased by 1.5 times. In particular, the per-capita PF for Russia showed substantial growth, increasing 17-fold from 2000 to 2013. Increases in these nations’ per-capita PF were mainly driven by their food consumption, which is becoming increasingly westernized^3^. The per-capita PF of Switzerland, Greece, Ireland and the United States were more likely to be boosted by the consumption of chemical products, and those of Luxemburg, Germany and Belgium tended to be increased by consumptions of coke and refined petroleum products, rather than food. To reduce these PFs induced by household consumption, promoting the consumers’ awareness of the threats posed by palm oil consumption is essential, as are improvements in food, chemical products, and biodiesel production technologies. The LUC footprints estimated in this study indicate which nations are major contributors to the environmental impacts triggered by palm oil production. Since these LUC footprints are based on spatial data that consider geographical changes in the land use associated with palm oil plantations^38^, it potentially allowed for extending the assessment to other environmental impact footprints. For example, the spatial environmental impact data as biodiversity loss^45^ and air pollution^32^ could be combined with fire area data as proposed in this study. It is considered that ecolabeling information that clarifies the extent to which the consumers have threatened the environment would be an effective demand-side policy^34,46^.

Interestingly, the share of domestic Indonesian consumption compared to the global Indonesian PF decreased from 17% in 2000 to 11% in 2013; however, at a per-capita level, the Indonesian PF is not small compared to other nations. As estimated above, the per-capita PF of Indonesian household consumption in 2013 was between those of Switzerland and Germany, with Indonesia ranked eighth in terms of per-capita PF in the world. Palm oil production in Indonesia is therefore essential for stabilizing, not only their economy through exports, but also their lifestyles. Despite good economic returns of oil palm cultivation for the farmers, oil palm cultivation has been shown to increase economic inequality among farmers^8,47,48^. Farmers experienced a boost in income in response to the oil palm boom, but this increase in income was limited to farmers who own farmland. Farmers with little or no farmland are typically hired by oil palm plantations or mills as casual workers. While the oil palm industry increased the demand for agricultural labor, the economic benefits to these workers is low. Several studies reported that workers in plantations and mills were often paid less than the minimum wage and are frequently exposed to dangerous chemical processes^49,50^. In addition, ecosystem degradation, including the deforestation, has negative impacts on social outcomes in Indonesia, including agricultural production^51^, fishing yield^52^, and human health^53^. In this context, understanding how and who is affected by the palm oil industry is essential for providing a safety net and for meeting the aims of the SDGs^21^. It is therefore necessary to implement alternative remediatory measures in order to balance economic gains and mitigate aggressive exports of palm oil given the impact of the latter on environmental deterioration and negative socioeconomic outcomes in Indonesia. As the number of RSPO ratifications has increased in recent years, building on financial support schemes based on consumption-based accounting methods, as described in this study, could promote protections for both ecosystems and society.

Finally, we recognize that a more detailed dataset containing information related to the global supply chains, palm oil production and consumption, and impact indicators will enrich the abovementioned discussion. Higher sectoral resolution for the PF is essential for implementing the labeling and taxation measures described above, because it is not yet clear which food or chemical products have a substantial contribution to the PF and associated environmental impacts. Further, it would be advantageous to visualize the linkages between palm oil consumption and the production sites where the LUC and forest fires occur for policymaking purposes^54–56^. These technical advances require the development of another detailed dataset containing global and local supply chains and environmental impact information. Through overcoming these challenges, as well as the other limitations detailed in the supporting information (SI), it may be possible to help policymakers understand the connection between their activities and the issues that arise outside of their nations. Appreciating these connections may facilitate the development of policy schemes required for either restricting or taxing consumption, not only to reduce dependencies on the palm oil but also to protect the local farmers in Indonesia.

## Methodology

### Estimation of the international palm oil and palm kernel flows

This study used material flow analysis (MFA) to quantify the international flows of Indonesian palm oil. Both palm oil (PO) and palm kernel oil (PKO) production and consumption for each nation were obtained from the FAOSTAT Crop Primary Equivalent^57^. The available range of these data is from 1986 to 2013. The crop primary equivalent records the physical amounts (ton; t) of production, import and export quantities, stock variation, processing, food supply quantity, other uses (i.e. non-food supply, such as for soap), feed, losses, and domestic supply quantity for 236 countries and regions. However, the Crop Primary Equivalent does not provide us with the trade flows between nations; i.e., where PO and PKO are imported to and exported from. We therefore linked the above normalized information with the Detailed Trade Matrices^6^, which list the import and export nations for both PO and the PKO in both monetary and quantitative terms. We created a concordance table based on export matrices to estimate the quantities of PO and PKO between nations because the amount of trade information that is available for exports is higher than that of imports. The detailed methods of estimating the global PO and PKO flows are described in the supporting information (SI).

### Quantification of indirect palm oil consumption induced by national final demands

To quantify the palm oil consumption for national final demands (i.e. palm oil footprint), this study utilized an input–output analysis with multi-regional input–output table (MRIO). An MRIO describes the monetary transactions across the economic sectors in nations and regions, and has been increasingly used as a method for quantifying the ultimate requirements for the final demands of a nation within the context of the international supply chains^58^. This study adopted the hybrid IO technique to quantify the embodied PO and PKO for a nation^59^.

This study also adopted the World Input–Output Database (WIOD) for retrieving the global MRIO data for the period 2000 to 2013^41^. The WIOD is comprised of 56 commodity sectors across the 43 nations and the rest of the world (ROW), and has been published during the period 2000 to 2014. Here, the production and consumption of PO and PKO is considered to be aggregated into the sector *crop and animal production, hunting and related service activities* of the WIOD. This study performed sectoral disaggregation to obtain the new commodity sector for PO and PKO, which we referred to as the “crude palm oil production” sector from *crop and animal production, hunting and related service activities*. In addition, a hybrid (quantitative and monetary) model based on the WIOD was also compiled. Further, we allocated the material flows of both PO and PKO for food supply quantity, non-food uses, processing, and feed to the following commodity sectors: *crop and animal production, hunting and related service activities* attributed to feed, *manufacturing of food products, beverages and tobacco products* which was attributed to food and processing, and *manufacturing of chemicals and chemical products*, *manufacture of coke and refined petroleum products*, and *electricity, gas, steam and air conditioning supply*, which was attributed to non-food, respectively. Note that the elements on the vertical direction of the crude palm oil production sector are all zero because we could not obtain the intermediate inputs from the other commodity sectors. Although we did not focus on the upstream supply chains of the PO and PKO produced in Indonesia in this study, doing so would lead to underestimating the results due to excluding the indirect palm oil sources used to produce them. Further, because the inputs of PO and PKO are not available for the three non-food sectors, the intermediates were predicted by multiplying the amounts of PO and PKO by the sum of the ratios of the intermediate outputs of these three sectors.

Based on our hybrid model, the footprint for PO and PKO (i.e. PF) produced by nation *r* could be estimated using Eq. (1).

1 $$ PF_{i}^{r} = \mathop \sum \limits_{s} \mathop \sum \limits_{j} l_{ij}^{rs ^{\prime}}f_{j}^{s^{\prime}} $$

where $l_{ij}^{rs^\prime}$ denotes the element of the Leontief inverse matrix in the *i*th row of the hybridized IO table when commodity *i* is attributed to item *k*. $f_{j}^{s^\prime}$ denotes the element of the final demand vector for nation *s* in the hybridized IO. Note that PO and PKO are not consumed directly in the final demand sectors because this study assumes that all of the PO and PKO are utilized as intermediates to produce final products via *manufacturing of food products*, *beverages and tobacco products* (for food), *manufacturing of chemicals and chemical products* (for non-food items such as soaps, grease, and cosmetics), and *manufacture of coke and refined petroleum products* (for biodiesel).

### Estimations of contributions of each nation to the land use changes and resultant fires associated with oil palm plantation in Indonesia

To estimate the LUC in Indonesia associated with the PF, we estimated the size of the areas that were converted to oil palm plantations from Austin et al.^38^. These authors highlighted both the size of areas and produced maps of the large-scale oil palm plantations in the major regions of Indonesia; i.e. Sumatra, Kalimantan, and Papua at five-year intervals during 1995–2015. The maps, which were created based on the Landsat composites and national data on land cover provided by the Ministry of Environment and Forestry, were very similar to those showing forest cover loss that were produced in a similar study^60^. The areas that were converted to oil palm plantations were not only classified as forest, but also non-forest areas such as swamps, scrubland, savannah, agriculture, and timber plantation.

In this study, palm oil consumption in the analytical year is assumed to promote the establishment of additional palm oil plantations that are adjacent to existing plantations. In other words, we estimated how much the associated environmental impacts were driven by an increase in the PF during the analysis period using Eq. (2).

2 $$ E^{rs} (t) = \frac{{\Delta_{(t)} E^{r} }}{{\Delta_{(t)} (\mathop \sum \nolimits_{s} PF^{rs} )}}\Delta_{(t)} PF^{rs} $$

where $E^{r}$ represents the total environmental impacts associated with palm oil production in nation *r*. In this study, the LUC estimated by Eq. (2) is referred to as the LUC footprints. *t* denotes the analytical period from *t*_0_ to *t*_1_ (e.g., 1995–2000). $\Delta_{(t)}$ denotes the cumulative values of interest during the period. Due to the data constraints, these impact footprints were estimated in two periods; between 2000–2005 and between 2005–2010. Further, the per-capita impact footprints for the nations were quantified by dividing $E^{rs} (t)$, obtained from Eq. (2), by their cumulative populations during each of the periods. The national populations were obtained from FAOSTAT.

In addition, to demonstrate the linkage between the LUC and the other environmental indicators, we first attempted to estimate the contribution of each nation to the areas of fires considered to be associated with the plantation development for palm oil production in Indonesia using spatial data of oil palm plantations and the occurrence of fires. The areas of fires that occurred in Indonesia were retrieved from the Global Fire Atlas with Characteristics of Individual Fires database maintained by NASA's Oak Ridge National Laboratory Distributed Active Archive Center^61^. The Global Fire Atlas is a global dataset that tracks the day-to-day dynamics of individual fires to show the timing and location of ignitions, fire size, duration, daily expansion, fire line length, speed, and direction of spread. These individual fire characteristics were derived based on the Global Fire Atlas algorithm which estimated day of burn at 500 m resolution using the Moderate Resolution Imaging Spectroradiometer (MODIS) Collection 6 MCD64A1 burned area product. The algorithm identified 13.3 million individual fires (≥ 21 ha or 0.21 km^2^; the size of one MODIS pixel) over 2003–2016^39^.

By combining these two databases of the LUC and the fires, we estimated the area of fires associated with plantation development, as shown in Supplementary Fig. S1 (in the SI). Fire location associated with plantation development in this study was defined as an area where fires occurred at a site that was converted to a new plantation within five years of the analyzed period. The size of the new plantation was determined by measuring the differences in the plantation areas during the analysis period. From the map and database of Global Fire Atlas, we aggregated individual day-to-day fires into annual fires and recorded their locations. Finally, the areas of fires associated with plantation development were estimated by calculating the overlaid locations of fires and their areas with the map of oil palm plantations provided by Austin et al.^38^. The analysis period was only 2005–2010 because, although the map of the oil palm plantations was for the period 1995–2015, forest fire data were only available after 2002 and the PF data until 2013. We used ArcGIS 10.5.1 for the calculations.

## Supplementary information

Supplementary Information.
