# Supplementary material for: Trends in global dependency on the Indonesian palm oil and resultant environmental impacts
Source: Sci Rep. 2020 Nov 26;10:20624. doi: 10.1038/s41598-020-77458-4 (PMC7692496; doi:10.1038/s41598-020-77458-4)
Supplement: Supplementary file 1 — Supplementary Information. [file 41598_2020_77458_MOESM1_ESM.pdf]

Supporting Information

Trends in Global Dependency on the Indonesian Palm Oil and Resultant Environmental Impacts

Yosuke Shigetomi[1*], Yuichi Ishimura[2], Yuki Yamamoto[1]

[1]Faculty of Environmental Science, Nagasaki University, 1-14 Bunkyo-machi, Nagasaki 852-8521, Japan

[2]Faculty of Economics, Kindai University, 3-4-1 Kowakae, Higashi Osaka, Osaka 577-8502, Japan

*Corresponding author: Yosuke Shigetomi, <y-shigetomi@nagasaki-u.ac.jp>

Compilation of the international palm oil flows using FAOSTAT

This study used material flow analysis (MFA) to quantify the international flows of Indonesian palm oil. Both palm oil (PO) and palm kernel oil (PKO) production and consumption for each nation were obtained from the FAOSTAT Crop Primary Equivalent [1]. The available range of these data is from 1986 to 2013. The crop primary equivalent records the physical amounts (ton; t) of production, import and export quantities, stock variation, processing, food supply quantity, other uses (i.e. non-food supply, such as for soap), feed, losses, and domestic supply quantity for 236 countries and regions. The summation of production, import quantity, and stock variation is considered to be consistent with the sum of export quantity and domestic supply quantity as expressed by equation (1). In addition, the domestic supply quantity should be equal to the sum of processing, food supply quantity, other uses, feed, and losses as presented by equation (2).

$$P + IM + V = D + EX \quad (1)$$

$$D = \mu + \pi + \rho + \sigma + \varphi \quad (2)$$

where *P*, *IM*, *V*, *D*, and *EX* denote the production, import quantity, stock variation, export quantity, and total domestic supply quantity. μ, π, ρ, σ, and φ represent the domestic supply quantities by supply category, i.e., processing, food supply quantity, other uses, feed, and losses. However, these

material balances are not satisfied in many of the recorded countries and regions in the dataset. In addition, since the values of domestic supply quantities for each category were negative due to a statistical error, the production and consumption flows were normalized as follows. In case either $\mu,\ \pi,\ \rho,\ \sigma$, or φ takes a negative value, it was replaced with 0. Next, if the balance equation (1) was not satisfied in the country of interest, then the normalized total domestic supply quantity, $\bar{D}_k^r$, was determined as shown in equation (3).

$$\bar{D}_k^r = D_k^r - (P_k^r + IM_k^r + V_k^r - EX_k^r) \tag{3}$$

where the superscript denotes the country of interest while subscript *k* indicates whether the product is PO or PKO. In this equation, if the balance equation (1) was not satisfied in the country of interest, then $\bar{D}_k^r = D_k^r$ (i.e. $P_k^r + IM_k^r + V_k^r - EX_k^r = 0$) was satisfied. Next, if the balance equation (2) was not satisfied in the country of interest, then $\mu_k^r,\ \pi_k^r,\ \rho_k^r$, and σ_k^r were normalized using the ratio of the normalized domestic supply quantity to the sum of processing, food supply quantity, other uses, feed, and losses in their raw values, respectively. Equation (4) shows the procedure used for obtaining the normalized μ_k^r.

$$\bar{\mu}_k^r = \mu_k^r \frac{\bar{D}_k^r}{D_k^r} \tag{4}$$

$\bar{\mu}_k^r,\ \bar{\pi}_k^r,\ \bar{\rho}_k^r$, and $\bar{\sigma}_k^r$ (i.e. the normalized $\pi_k^r,\ \rho_k^r$, and σ_k^r) were obtained in the same manner of $\bar{\mu}_k^r$ in equation (4).

For some of the nations, either the sum of $\mu_k^r,\ \pi_k^r,\ \rho_k^r$, and σ_k^r, or D_k^r are all zero or all negative (e.g., the domestic supply quantities of palm oil in Argentina in 2000). This may be because of statistical errors, which meant that equations (3) and (4) could not be used for normalization. We therefore attempted to adjust $\mu_k^r,\ \pi_k^r,\ \rho_k^r$, and σ_k^r, or D_k^r by using the stock variation (V_k^r) and export quantity (EX_k^r) for such the nations. When the sum of $\bar{\mu}_k^r,\ \bar{\pi}_k^r,\ \bar{\rho}_k^r$, and $\bar{\sigma}_k^r$ is zero or $\bar{D}_k^r < 0$, the adjusted stock variation $\bar{V}_k^r$ and export quantity $\overline{EX}_k^r$ were determined using equations (5)–(7).

$$\bar{D}_k^r = \bar{\mu}_k^r + \bar{\pi}_k^r + \bar{\rho}_k^r + \bar{\sigma}_k^r + \bar{\varphi}_k^r \tag{5}$$

$$\bar{V}_k^r = -\bar{D}_k^r \tag{6}$$

$$\overline{EX}_k^r = EX_k^r - \bar{D}_k^r \tag{7}$$

In the above procedure, the input-output information for PO and PKO are identified. However, the Crop Primary Equivalent does not provide us with the trade flows between nations;

i.e., where PO and PKO are imported to and exported from. We therefore linked the above normalized information with the Detailed Trade Matrices [2], which list the import and export nations for both PO and the PKO in both monetary and quantitative terms. There are two ways to make the concordance tables that are used to map the trade nations and quantities of PO and PKO based on the table describing the import and/or export quantities. Although the information for imports is considered to be more accurate than that of exports [3], we created a concordance table based on export matrices to estimate the quantities of PO and PKO between nations because the amount of trade information that is available for exports is higher than that of imports. We therefore determined the PO and PKO flows from nation *r* to nation *s* using the concordance table using equations (8) and (9).

$$\mu_k^{rs} = \theta_k^{rs} \overline{IM}_k^s \frac{\bar{\mu}_k^s}{\bar{D}_k^s} \ (r \neq s) \tag{8}$$

$$\theta_k^{rs} = \begin{cases} \dfrac{e_k^{rs}}{\sum_s e_k^{rs}} & \left(\sum_s e_k^{rs} \neq 0\right) \\ 0 & \left(\sum_s e_k^{rs} = 0\right) \end{cases} \tag{9}$$

where e_k^{rs} denotes the amount of palm oil imported by nation *s* from nation *r* in the Detailed Trade Matrices. θ_k^{rs} represents the relative amounts of PO and PKO that are imported by nation *r*. The rest of π_k^{rs}, ρ_k^{rs}, and σ_k^{rs} were made in the same manner as μ_k^{rs}. Note that we assume that nation *s* imports the PO and PKO from nation *r* with respect to their commodity balance, because there is no data available for how each of the commodities were produced in nation *r* and consumed in nation *s*.

Finally, the consumption amounts of PO and PKO produced domestically (i.e. *r*=*s*) were quantified using equation (10).

$$\mu_k^{rr} = (\bar{P}_k^r + \bar{V}_k^r) \frac{\bar{\mu}_k^r + \frac{\varphi_k^r}{4}}{\bar{D}_k^r} \ (r = s) \tag{10}$$

Because it is impossible to quantify precisely where the amounts of PO and PKO that are lost, we allocated φ_k^r equally to the rest of π_k^r, ρ_k^r, and σ_k^r. In equation (10), $\varphi_k^r/4$ represents the amounts of PO and PKO lost in each process in all of the supply categories (e.g., losses due to transportation).

Methodological limitations

There remain limitations and uncertainties regarding the methodologies and datasets employed in this study. First, we were unable to trace the detailed allocations of the palm oil (i.e. PO and PKO) for other uses (i.e. non-food) on the crop primary equivalent to the related industrial sectors in the WIOD for each nation. In this study, as mentioned above, the PO and PKO were allocated to the three non-food sectors related to palm oil (i.e. chemicals, petroleum, and electricity) based on the size of the monetary output of these sectors on the WIOD. This assumption may affect the uncertainty of non-food PF results. Second, we did not consider the differences in consumption patterns of domestically produced palm oil and palm oil imported into each nation. For example, Indonesia imports small amounts of palm oil from Malaysia, but it was not possible to distinguish how much of the imported the palm oil and the domestic the palm oil were consumed across food and non-food sectors. Further, it was not possible to avoid double counting in the food and non-food sectors in the hybridized IO model because the price information of the palm oil used to convert the physical quantities consumed by each sector into monetary amounts was inconsistent. Instead, to overcome this limitation, we confirmed that considering the deduction of monetary amounts for the palm oil seldom affects the PF results. We did this by calculating the variance in PF using the provisional palm oil prices that should be deducted up to 99% of the monetary amounts for the sectors consuming the palm oil.

The LUC which comprises the land footprint relies on a previous study that focuses solely on the areas that have been developed as the large palm plantations in Sumatra, Kalimantan, and Papua in Indonesia [4]. Nevertheless, the total LUC during the studied periods are almost consistent with official Indonesian statistics, which considered total changes in areas related to palm oil production [5]. Hence, it is considered that the results obtained in this study are compatible with other studies that consider total Indonesian palm oil production.

Detected areas of fires associated with oil palm plantation in Indonesia

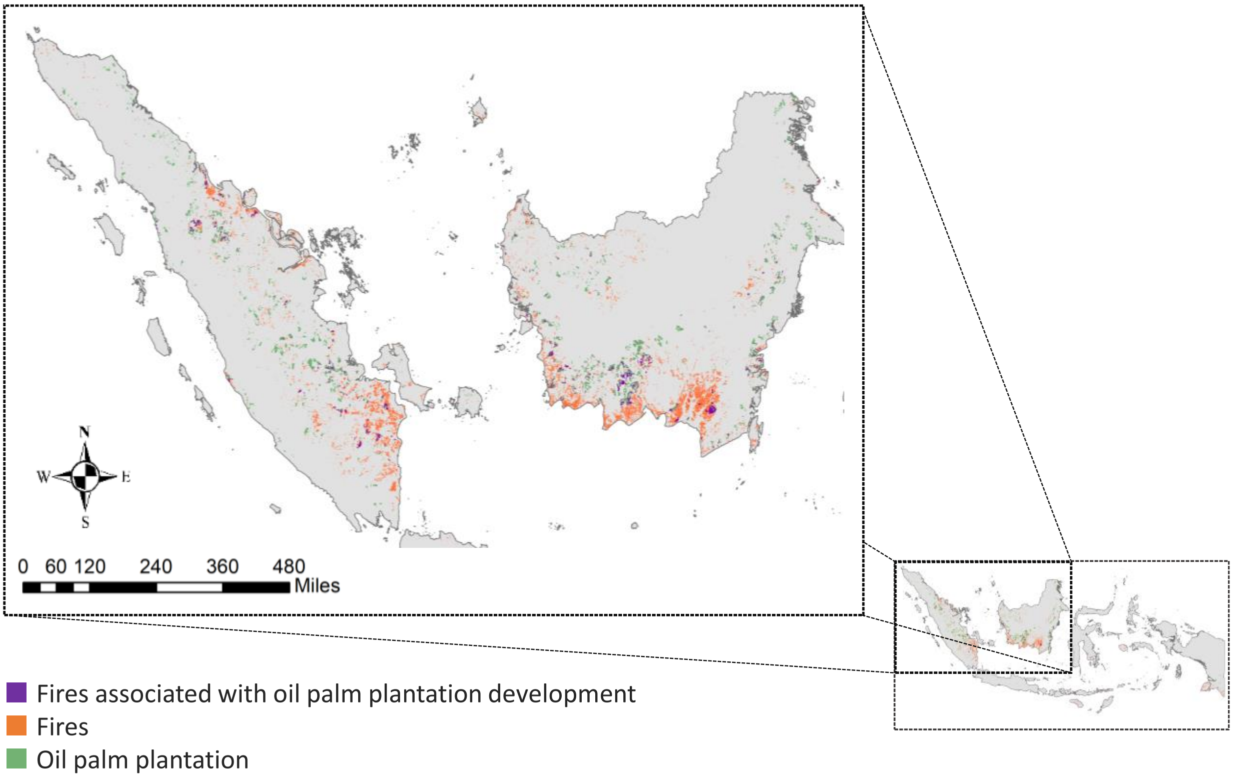

Figure S1 Areas of fires associated with oil palm plantation (purple), fires outside oil palm plantations (orange), oil palm plantations (green) from 2005 to 2010 in Indonesia. Only Sumatra and Kalimantan are shown (inset) because fires were restricted to these areas. The figure is created by using ArcGIS 10.5.1 (https://www.esrij.com/). Geographical data based on the GADM database 2.8 (https://www.gadm.org/).

References

1. FAO. Commodity Balances - Crops Primary Equivalent. (2018). Available at: http://www.fao.org/faostat/en/#data/BC.
2. FAO. Detailed trade matrix. (2019). http://www.fao.org/faostat/en/#data/TM
3. World Bank. World Integrated Trade Solution (WITS). (2010). Available at: https://wits.worldbank.org/wits/wits/witshelp/Content/Data_Retrieval/T/Intro/B2.Imports_Exports_and_Mirror.htm.
4. Austin, K. G. *et al.* Shifting patterns of oil palm driven deforestation in Indonesia and implications for zero-deforestation commitments. *Land use policy* **69**, 41–48 (2017).

5. Directorate General of Plantation. *Statistik Perkebunan Indonesia 2014-2016: Kelapa sawit*. *Tree Crop Estate Statistics of Indonesia* (2015).
